# The Effect of SIRT3/Ac-SOD2 Mediated Oxidative Stress and HCN1 Channel Activity on Anesthesia/Surgery Induced Anxiety-Like Behavior in Mice

**DOI:** 10.3389/fmed.2022.783931

**Published:** 2022-03-15

**Authors:** Hui-Hui Miao, Qiang Liu, Ning Wang, Yan-Ping Liu, Chen Chen, Hai-Bi Wang, Hui Huang, Wei-Feng Wu, Jia-Tao Lin, Yong-Kang Qiu, Chuan-Wu Zhang, Cheng-Hua Zhou, Yu-Qing Wu

**Affiliations:** ^1^Department of Anesthesiology, Beijing Shijitan Hospital, Capital Medical University, Beijing, China; ^2^Jiangsu Province Key Laboratory of Anesthesiology, Xuzhou Medical University, Xuzhou, China; ^3^Jiangsu Key Laboratory of New Drug Research and Clinical Pharmacy, Xuzhou Medical University, Xuzhou, China; ^4^NMPA Key Laboratory for Research and Evaluation of Narcotic and Psychotropic Drugs, Xuzhou Medical University, Xuzhou, China

**Keywords:** anxiety, anesthesia/surgery, SIRT3, SOD2, HCN1

## Abstract

Anxiety disorders are the most common psychiatric diseases, and perioperative factors often increase the incidence of anxiety. However, the mechanism and treatment for perioperative anxiety, especially anesthesia/surgery-induced postoperative anxiety, are largely unknown. Sirtuin 3 (SIRT3) which located in the mitochondria is the NAD-dependent deacetylase protein. SIRT3 mediated oxidative stress is associated with several neuropsychiatric diseases. In addition, hyperpolarization-activated cyclic nucleotide-gated 1 (HCN1) channel is also reported involved in anxiety symptoms. The purpose was to assess the role of SIRT3 on postoperative anxiety like behavior in C57/BL6 mice. We found that SIRT3 level reduced and HCN1 expression level increased in mice medial prefrontal cortex (mPFC) as well as anxiety like behavior postoperatively. In interventional research, SIRT3 adeno-associated virus vector or control vector was injected into the mPFC brain region. Enzyme-linked immunosorbent assay, immunofluorescence staining, and western blotting were employed to detect oxidative stress reactions and HCN1 channel activity. SIRT3 overexpression attenuated postoperative anxiety in mice. Superoxide dismutase 2 (SOD2) acetylation levels, SOD2 oxidative stress activity, mitochondrial membrane potential levels, and HCN1 channels were also inhibited by SIRT3 overexpression. Furthermore, the HCN1 channel inhibitor ZD7288 significantly protected against anesthesia/surgery-induced anxiety, but without SIRT3/ac-SOD2 expression or oxidative stress changes. Our results suggest that SIRT3 may achieve antianxiety effects through regulation of SOD2 acetylation-mediated oxidative stress and HCN1 channels in the mPFC, further strengthening the therapeutic potential of targeting SIRT3 for anesthesia/surgery-induced anxiety-like behavior.

## Introduction

Anxiety is a state of restlessness and worry (1). The perioperative period is usually associated with increased anxiety in patients (2). Compared to preoperative anxiety, postoperative anxiety has not received much attention, even though researchers have suggested that postoperative anxiety may lead to adverse outcomes (3). Furthermore, the mechanism and treatment of anxiety are unclear (4–8). Medical therapy with the most widely used antianxiety sedatives (benzodiazepines) may not be suitable for postoperative anxiety because of side effects such as respiratory depression and delirium (9). Therefore, ruling out the mechanism of anxiety triggered by anesthesia/surgery is important for identifying new therapeutic targets.

Hyperpolarization-activated cyclic nucleotide-gated (HCN) ion channels, including HCN1–4 channels, may modulate the excitability of neurons (10). Several studies have pointed out that inhibited HCN1 channels exhibit anxiolytic effects (11–13). For example, in the hippocampus, HCN1 channel activity appears to induce anxiety (14). Moreover, in the prefrontal cortex, the HCN1 expression is predominantly among HCN1-4 (10). In the mouse brain, the medial prefrontal cortex (mPFC) is a critical region for anxiety emotions (15). HCN channels are involved in spontaneous electrical activity in the heart and brain (16). The normal activity of HCN channels is affected under stress conditions. In sick sinus syndrome, HCN4 expression is maintained by mitochondrial thioredoxin 2 through oxidative stress (17). Cyclic adenosine monophosphate (cAMP) regulates HCN channel opening by binding to the cyclic nucleotide-binding domain (16). HCN channels are sensitive to changes in the energy availability (18). cAMP is mainly generated by adenosine 5′-triphosphate (ATP) through the catalytic transformation of adenylate cyclase III (19), suggesting that mitochondria is essential to HCN1 channels. Previous studies have found that ZD7288 can reduce oxygen consumption and protect mitochondrial ATP production in H9C2 cells, indicating that HCN1 channel activity is related to mitochondrial membrane potential (MMP) (20).

Sirtuin 3 (SIRT3) is a nicotinamide adenine dinucleotide (NAD)-dependent deacetylase that is chiefly distributed in mitochondria-rich organs and tissues, such as the brain and neurons, and regulates mitochondrial protein function through acetylation (21–24). SIRT3 plays a key role in regulating mitochondrial dysfunction. In a Ti ion damage osteoblast cell model, the expression levels of SIRT3 were decreased, and SIRT3 overexpression reduced superoxide dismutase 2 (SOD2) acetylation, increased SOD2 activity, and inhibited mitochondria-generated reactive oxygen species (ROS) production and cell viability (25). In a previous study, a synthesized rhamnoside derivative (PL171), as a SIRT3 agonist, suppressed MMP reduction and damage due to mitochondrial oxygen consumption stimulated by amyloid beta (Aβ)_42_ (26). In another study in PC12 cells, Aβ_1−40_ decreased cell viability, SIRT3 activity, and MMP levels and increased ROS production, while *Codonopsis pilosula* polysaccharides partially recovered ATP by increasing SIRT3 expression levels (27). However, there are limited data on the impact of SIRT3 on anesthesia/surgery-induced anxiety-like behavior of mice. Therefore, the relationship of SIRT3 on SOD2 acetylation, MMP reduction, and related HCN1 channel dysfunction in postoperative anxiety-like behavior and its underlying neuroprotective mechanism were studied in this research work.

## Materials and Methods

### Animals and Materials

The experimentations were performed at Xuzhou Medical University and permitted according to the local Animal Care and Use Committee. C57BL/6J mice (male, age: 3–4 months, body weight: 25–30 g) were bought from Nanjing University Model Animal Research Center. The mice were administrated with standard light/dark 12 h cycle and the house temperature around 22–25°C.

### Anesthesia/Surgery Treatment

We performed tibial fracture surgery in the mice under isoflurane anesthesia to mimic a clinical operation, as shown in a previous study (28). A construct was packaged into an adeno-associated virus (AAV)2/8 chimeric virus, pAAV-mNeonGreen, to overexpress SIRT3 protein. We also set a pAAV-mNeonGreen vector without SIRT3 as the control AAV vector. These viral construct procedures were performed by OBiO Technology. ZD7288 (1 μL/mL) was dissolved in H_2_O and microinjected (0.5 μL/side) into the mPFC of mice bilaterally. ZD7288 (4-ethylphenylamino-1,2-dimethyl-6-methylaminopyrimidinium chloride) was obtained from Sigma-Aldrich (Z3777, Sigma, USA). The mice were given 3.0% isoflurane during the induction period and followed by 1.5% isoflurane in maintenance period. The detailed procedures are described in our previous study (29). The mice were returned to their cage after they recovered from anesthesia/surgery. All mice received 2% lidocaine at the local incision site to relieve postoperative pain.

### Viral Microinjection

Viruses (titers >1.0 × 10^12^, 0.2 μL/side) were injected into the bilateral mPFC regions of the brain through stereotactic brain surgery according to the mouse brain atlas (anteroposterior +1.94 mm, lateral ± 0.30 mm, dorsoventral −2.50 mm). Virus injections were administered 4 weeks before the operation day. The location of viruse transfection were verified by immunofluorescence staining.

### Behavioral Tests

The open-field test (OFT) and elevated plus-maze (EPM) test were used to evaluate the anxiety level of the mice. In the OFT, the mice were placed on a white plastic open-field apparatus, with a size of 50 cm × 50 cm and divided into a grid of 8 × 8 squares. In the test session, the movement of the mice was recorded for 5 min. For anxiety assessment, the time each animal spent in the inner 6 × 6 cm square was evaluated. The percentage of time spent in the center of the box was used to measure the anxiety level of the mice and the duration of 5 min were selected according to some pervious reports (30–32). In EPM test, the mice were first placed in the experimental room for 1 day for habituation, followed by placing them in the middle of the maze. The percentage of time spent in the open arms was recorded and analyzed for anxiety level. Ethanol (75%) was used to clean the maze after each quiz and prevent olfactory cues.

### Western Blot Analysis

Western blotting was performed as described previously (33). The primary antibodies we used were as follows: HCN1 (1:2000, ab229340, Abcam, UK), SIRT3 (1:1000, 5490, Cell Signaling Technologies, USA), SOD2 (1:1000, 13141, Cell Signaling Technology, USA), ac-SOD2 (acetyl K68) (1:1000, ab137037, Abcam, UK). We used the ECL detection system (Beyotime Institute of Biotechnology, China) and ImageJ software for protein quantification.

### Immunofluorescence Analysis of HCN1

Immunofluorescence staining was used to study HCN1 channel activation. The heart perfusion was carried out with 0.9% saline in the mice,followed by 4% paraformaldehyde, in 0.1 M phosphate buffer (pH7.4). Then, as reported in our prior study, the brain tissues were removed quickly and postfixed in 4% paraformaldehyde overnight. Next step, it was cryoprotected in 30% sucrose. Frozen sections were performed then (VT1000S, Leica Microsystems). The coronal sections of the brain were cut consecutively at a thickness of 30 μm when the corpus callosum forceps was initially exposed and then the 10th sections were taken and stored in PBS. According to the Paxinos and Franklin's the Mouse Brain in Stereotaxic Coordinates, the positions of the coronal sections of the mPFC were approximately 1.65–1.75 mm anterior to the bregma. Goat serum (10%) was blocked for 1 h, then HCN1 antibody (1:100, ab84816, Abcam, UK) was incubated overnight at 4 °C. The secondary antibody was goat anti-mouse Alexa 594 (1:500, ab150080, Abcam), incubating for 2 h at room temperature in the dark. The fluorescence intensity was imaged with confocal microscope and used for analyzed (Zeiss, LSM880, Germany). 6 mice per group were used. The fluorescence intensity analysis methods were the same as those used in a previous study (29).

### SOD2 Activity Evaluation

Spectrophotometric assay kits were used to ascertain SOD2 activity (Elabscience, Wuhan, China).

### Determination of MMP

MMP was detected using JC-1 dye (Beyotime, Shanghai, China), as described previously (33). The samples were then incubated at 37 °C in a quainter. We used the Varioskan LUX microplate reader to determine the red (590 nm) and green (525 nm) fluorescence intensities.

### Statistical Analysis

GraphPad Prism 6.0 (GraphPad Software, Inc.) was used for statistical analyses. Data are shown as mean ± standard error of mean. Differences among the four groups were analyzed using one-way analysis of variance. Statistical significance was set at *p* < 0.05.

## Results

### SIRT3 Overexpression Protected Against Anesthesia/Surgery-Induced Anxiety-Like Behavior

**Figure 1:**
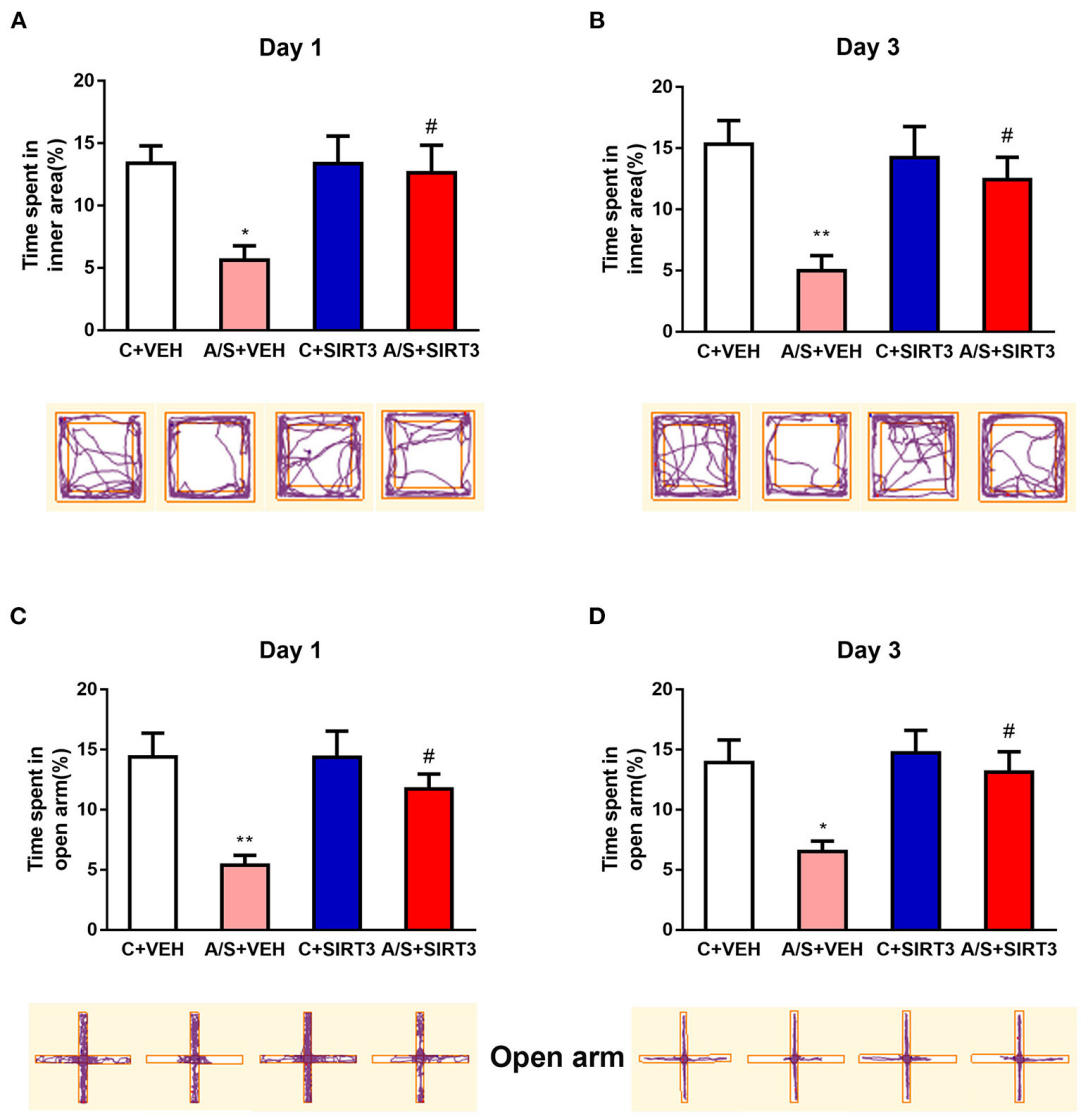
Effect of SIRT3 effect on anxiety-like behavior induced by anesthesia/surgery. **(A,B)** The percent time spent in inner area and movement trace of the OFT among the 4 groups. **(C,D)** The percent time in open arm and movement trace of the EPM among the 4 groups (*n* = 10 per group, *p < 0.05; **p < 0.01). Data are presented as the means ± SEM.

Previous studies have shown that anesthesia/surgery can induce adverse emotions (34, 35). To investigate whether anxiety-like behavior can be induced postoperatively, we employed the standard OFT and EPM test to trace the mice's movement among the four groups at different time points. In the OFT, the percentage of time that was spent in internal region in A/S + VEH group was significant decreased in comparison with C + VEH group at day 1 and day 3 postoperatively (*p* < 0.05, Figures 1A,B). Next, the percentage of time that was spent in EPM's open arm was also significant declined in A/S + VEH than C + VEH group at day 1 and day 3 after operation (^**^p < 0.01, ^*^p < 0.05, Figures 1C,D). The outcomes illustrated that anesthesia plus surgery might cause anxiety-like behavior in mice at post-operative period. Moreover, SIRT3 overexpression protected against the decreased percentage of time that was spent in OFT's interior region and the percentage of time that was spent in EPM's open arm in A/S + SIRT3 in comparison with group A/S + VEH at day 1/3 postoperatively. (# *p* < 0.05, Figures 1A–D). SIRT3 attenuated anesthesia/surgery induced anxiety emotion in mice was indicated by these data.

### SIRT3 Overexpression Meliorated Anesthesia/Surgery Induced SOD2 Acetylation in Mice mPFC

**Figure 2:**
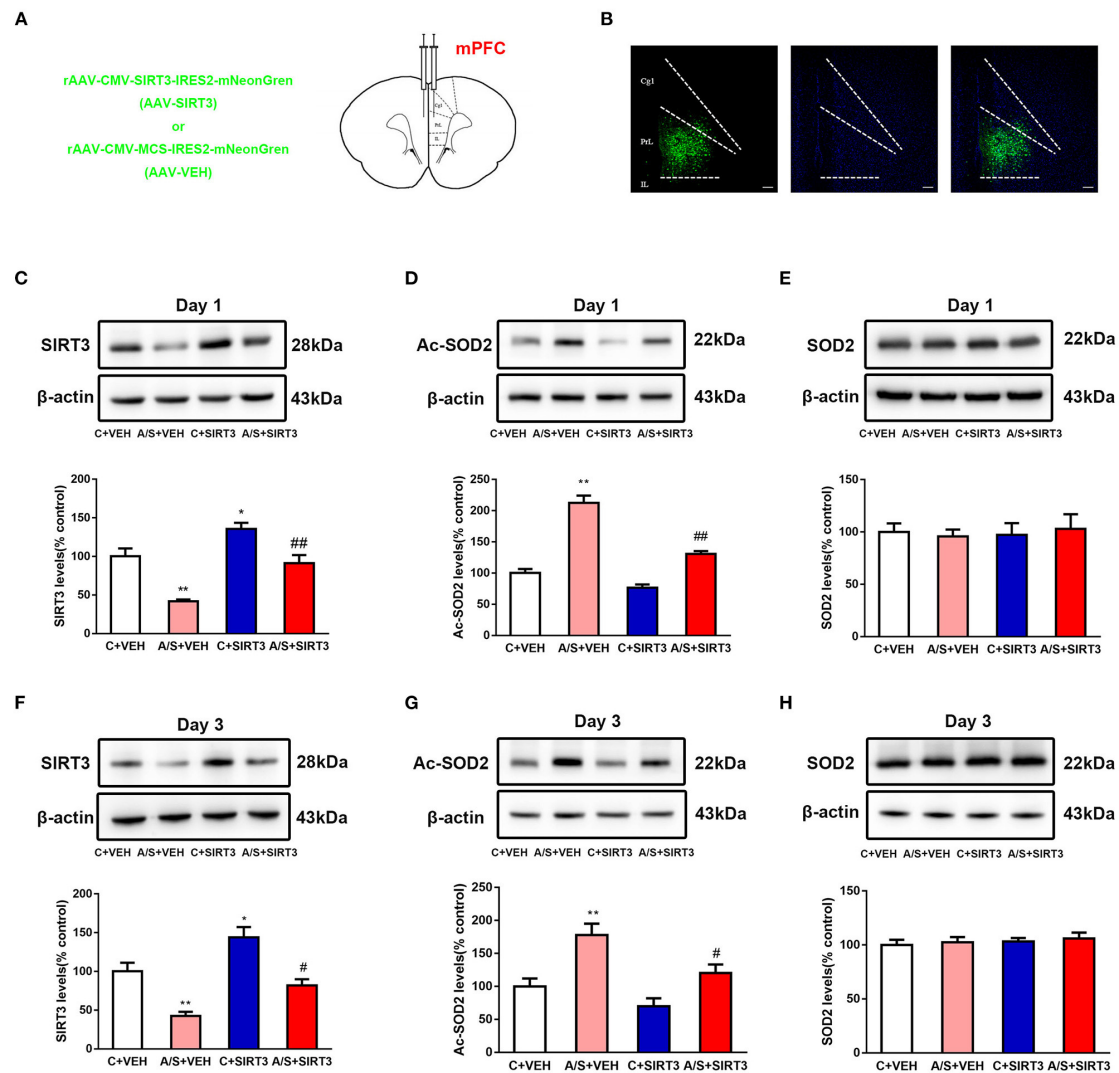
Effect of SIRT3 overexpression on SOD2/Ac-SOD2 levels. **(A)** Location of AAV-SIRT3 or AAV-VEH microinjection in the mPFC of mice. **(B)** AAV-SIRT3 vector expression in mice mPFC region is shown on fluorescence images. **(C-H)** SIRT3, Ac-SOD2, and SOD2 expression among the four groups on days 1 and 3 is shown by western blotting and quantification (*n* = 6 per group, *p < 0.05; **p < 0.01; ^#^*p* < 0.05; ^*##*^*p* < 0.01). Data are presented as the means ± SEM.

To reveal the function of SIRT3 in postoperatively anxiety, AVV-*SIRT3*-mNeonGreen was microinjected into the mPFC area, and AVV-mNeonGreen alone was used as control (Figures 2A,B). SIRT3 was raised in group C + SIRT3 but diminished in A/S + VEH in comparison with C + VEH group, while overexpress SIRT3 saved the diminished SIRT3 in group A/S + SIRT3 in comparison with group A/S + VEH (#*p* < 0.05, ^*^p < 0.05, ^**^p < 0.01, Figures 2C,F). Then, to demonstrate the substrate acetylation level of SIRT3, the ac-SOD2 K68 in mPFC was measured repeatedly among groups. As shown in Figures 2D,G, the ac-SOD2 K68 expression was increased significantly in A/S + VEH in comparison with C + VEH group, while the ac-SOD2 K68 was significantly diminished in A/S + SIRT3 in comparison with A/S + VEH group (#*p* < 0.05, ^**^p < 0.01). Meanwhile, we evaluated SOD2 K68 level in mPFC among groups at the same time point. There was not significantly difference of SOD2 level among 4 groups neither at day 1 or day 3 postoperatively (Figures 2E,H). These results suggested that SIRT3/ac-SOD2 signal pathway in mPFC was involved in anesthesia plus surgery caused anxiety-like behavior.

### SIRT3 Overexpression Attenuated Anesthesia/Surgery Related Oxidative Stress Reaction in Mice mPFC

**Figure 3:**
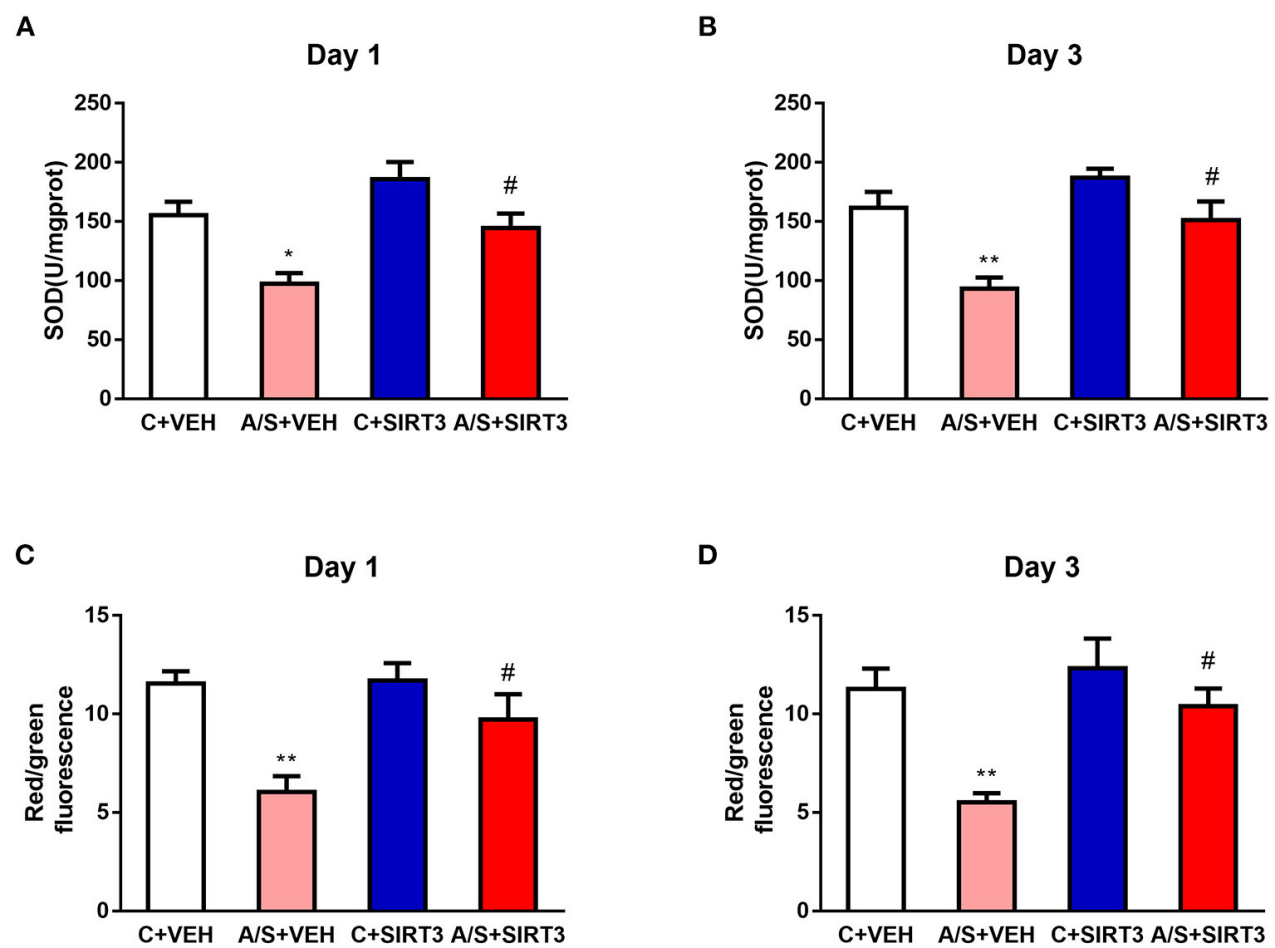
Effect of SIRT3 overexpression on mitochondrial oxidative levels. **(A–D)** Analysis of SOD2 and MMP in the mPFC region among the four groups (*n* = 6 per group, *p < 0.05; **p < 0.01; ^#^*p* < 0.05). Data are presented as the means ± SEM.

Evidence shows that SIRT3 plays an important role in mitochondrial function and that oxidative stress response is associated with surgical trauma (29, 33). Thus, we examined the effects of SIRT3 overexpression on MMP levels and SOD2 activity induced by anesthesia/surgery in the mPFC region of mice. Anesthesia/surgery induced decreased SOD2 activity and MMP in A/S + VEH in comparison with C + VEH group in mice mPFC region on day 1 and 3 postoperatively. Meanwhile, SIRT3 overexpression attenuated the decreased SOD2 activity and MMP level in A/S + SIRT3 in comparison with A/S + VEH group (#*p* < 0.05, ^*^p < 0.05, ^**^p < 0.01, Figures 3A–D). These consequences pointed out that mitochondrial oxidative stress reaction induced by anesthesia/surgery in mice mPFC was meliorated in the SIRT3 dependent way.

### HCN1 Channel Was Involved in the Neuroprotective Effect of SIRT3 Overexpression on Anxiety-Like Behavior Induced by Anesthesia/Surgery

**Figure 4:**
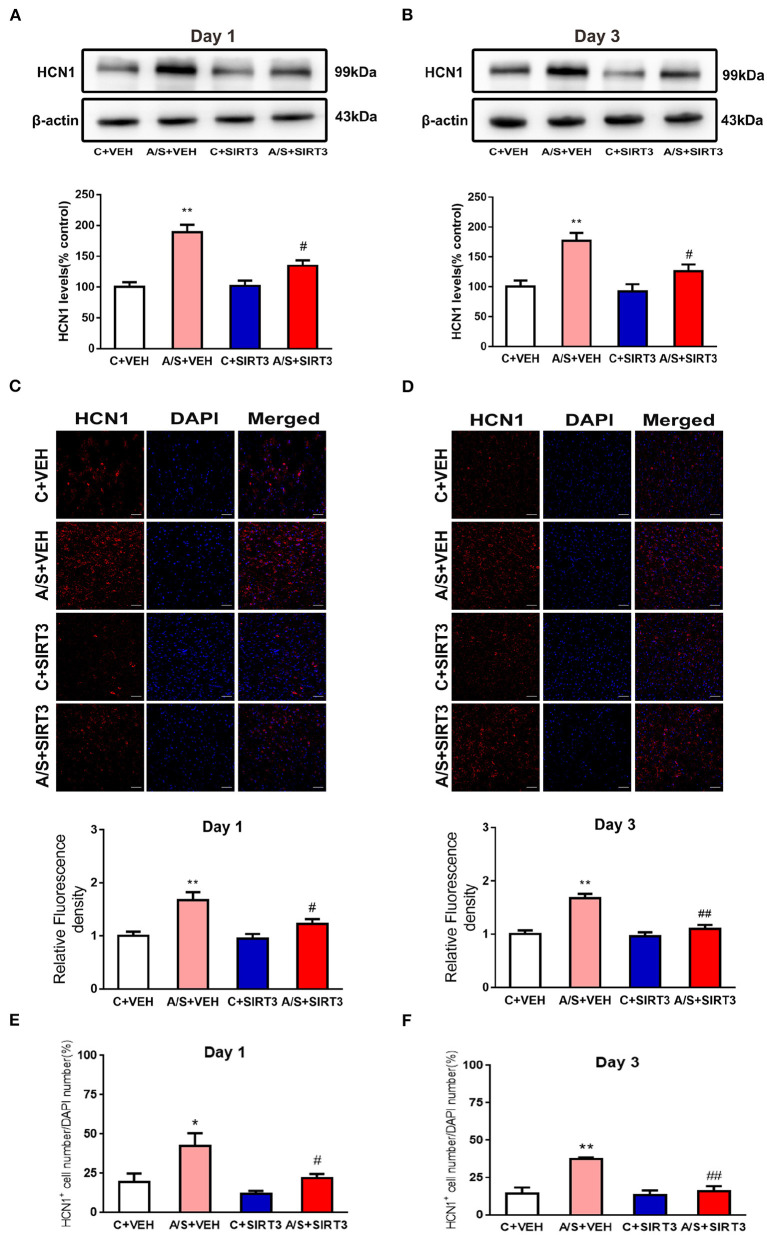
Effect of SIRT3 overexpression on the HCN1 channel. **(A,B)** Representative western blots and quantification of HCN1 levels in mPFC area. **(C–F)** Representative images and quantification of HCN1 fluorescence in mPFC area (*n* = 6 per group, *p < 0.05; **p < 0.01; ^#^*p* < 0.05; ^*##*^*p* < 0.01). Data are presented as the means ± SEM.

Several studies have reported that the HCN1 channel is related to anxiety in different disease models (11, 12, 14, 16, 36), but no studies have evaluated HCN1 channels in anesthesia/surgery-induced anxiety-like behavior and the dependence of HCN1 channel on SIRT3. Western blot showed that the HCN1 channel expression increased in group A/S + VEH in comparison with C + VEH group significantly, the HCN1 rise was meliorated by SIRT3 overexpression in A/S + SIRT3 in comparison with A/S + VEH group (#*p* < 0.05, ^**^p < 0.01, Figures 4A,B). Meanwhile, the immunofluorescence staining of HCN1 demonstrated that anesthesia/surgery induced HCN1 channel activated in A/S + VEH than in C + VEH group in the mPFC at day 1 and day 3 postoperatively. Additionally, HCN1 channel activation was inhibited by SIRT3 overexpression in group A/S + SIRT3 than A/S + VEH (##*p* < 0.01, #*p* < 0.05, ^**^p < 0.01, Figures 4C,D). Next, we analyzed the percentage of HCN1^+^ cell to DAPI as well. Anesthesia/surgery induced the number of cells that expressed HCN1 in A/S + VEH than in C + VEH group in the mPFC at day 1 and day 3 postoperatively. Accordingly, the percentage of HCN1^+^ cell was inhibited by SIRT3 overexpression in A/S + SIRT3 than A/S + VEH group (#*p* < 0.05, ^*^p < 0.05, Figures 4E,F). These consequences released that HCN1 channel might be involved in surgery trauma in SIRT3 dependent manner.

### ZD7288 Inhibited Anesthesia/Surgery-Induced HCN1 Channel Activation in Mice mPFC

**Figure 5:**
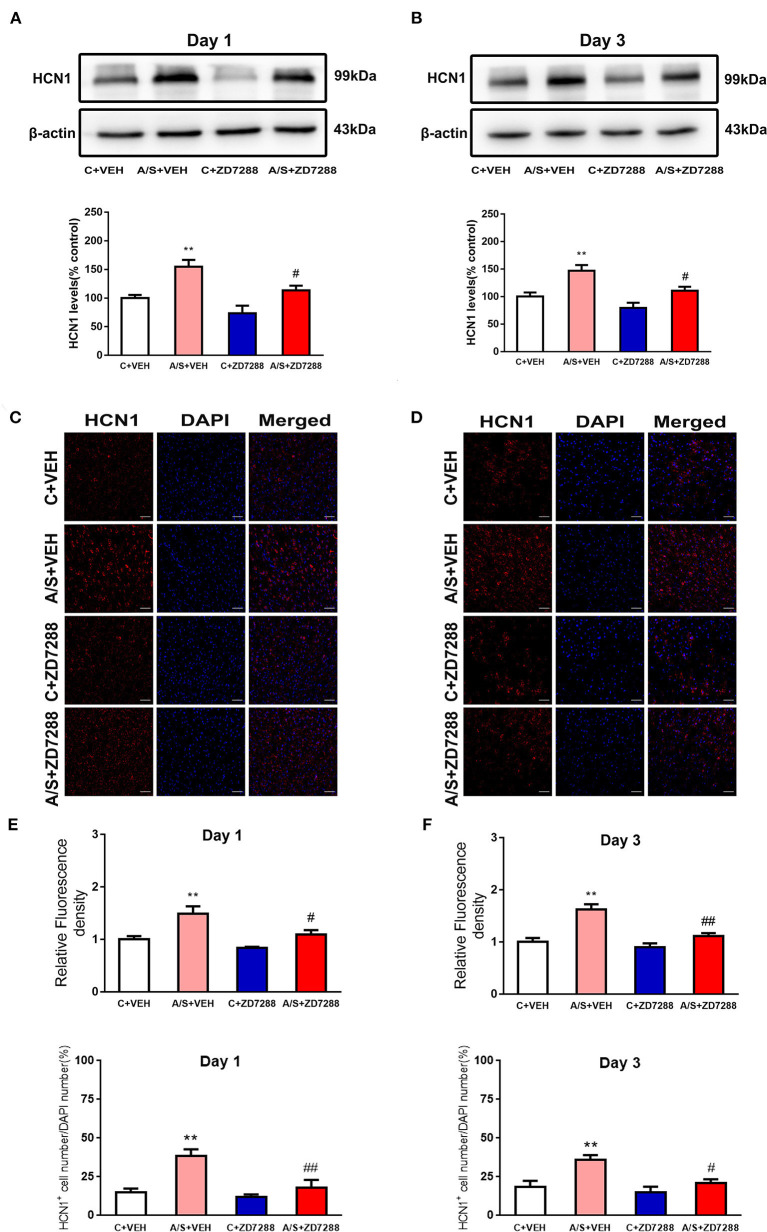
Effect of ZD7288 on the HCN1 channel. **(A,B)** Representative western blots and quantification of HCN1 levels in mPFC area. **(C–F)** Representative images and quantification of HCN1 fluorescence in mPFC area (*n* = 6 per group, *p < 0.05; **p < 0.01; ^#^*p* < 0.05; ^*##*^*p* < 0.01). Data are presented as the means ± SEM.

Recent studies have indicated that HCN1 channel activity induces anxiety in the hippocampus (14, 37). Indeed, blocking the HCN1 channel by ZD7288 has anxiolytic effects (13). Therefore, the HCN1 channel blocker ZD7288 was microinjected and HCN1 expression was measured by western blot and immunofluorescence staining. As shown in Figures 5A,B, HCN1 expression was significantly increased in group A/S + VEH day 1 and 3 after anesthesia/surgery (#*p* < 0.05, ^**^p < 0.01, Figures 5A,B). Additionally, the HCN1 expression was decreased by ZD7288 significantly in A/S + ZD7288 group than in group A/S + VEH postoperatively. Meanwhile, the immunofluorescence staining of HCN1 demonstrated that anesthesia/surgery induced HCN1 channel activated in A/S + VEH than in C + VEH group in the mPFC at day 1 and day 3 postoperatively. In Addition, HCN1 channel activation was inhibited in group A/S + ZD7288 (##*p* < 0.01, #*p* < 0.05, ^**^p < 0.01, Figures 5C,D). We also found that anesthesia/surgery induced the increase of the percentage of HCN1^+^ cell in A/S + VEH than in C + VEH group in the mPFC at day 1 and day 3 postoperatively. Accordingly, the percentage of HCN1^+^ cell was inhibited in A/S + ZD7288 group than A/S + VEH group (##*p* < 0.01, #*p* < 0.05, ^**^p < 0.01, Figures 5E,F). These consequences released that HCN1 channel activation in mice mPFC might be blocked by ZD7288 after anesthesia/surgery.

### Inhibition of the HCN1 Channel Did Not Affect Anesthesia/Surgery-Induced SIRT3 Decrease and SOD2 Acetylation in Mice mPFC

**Figure 6:**
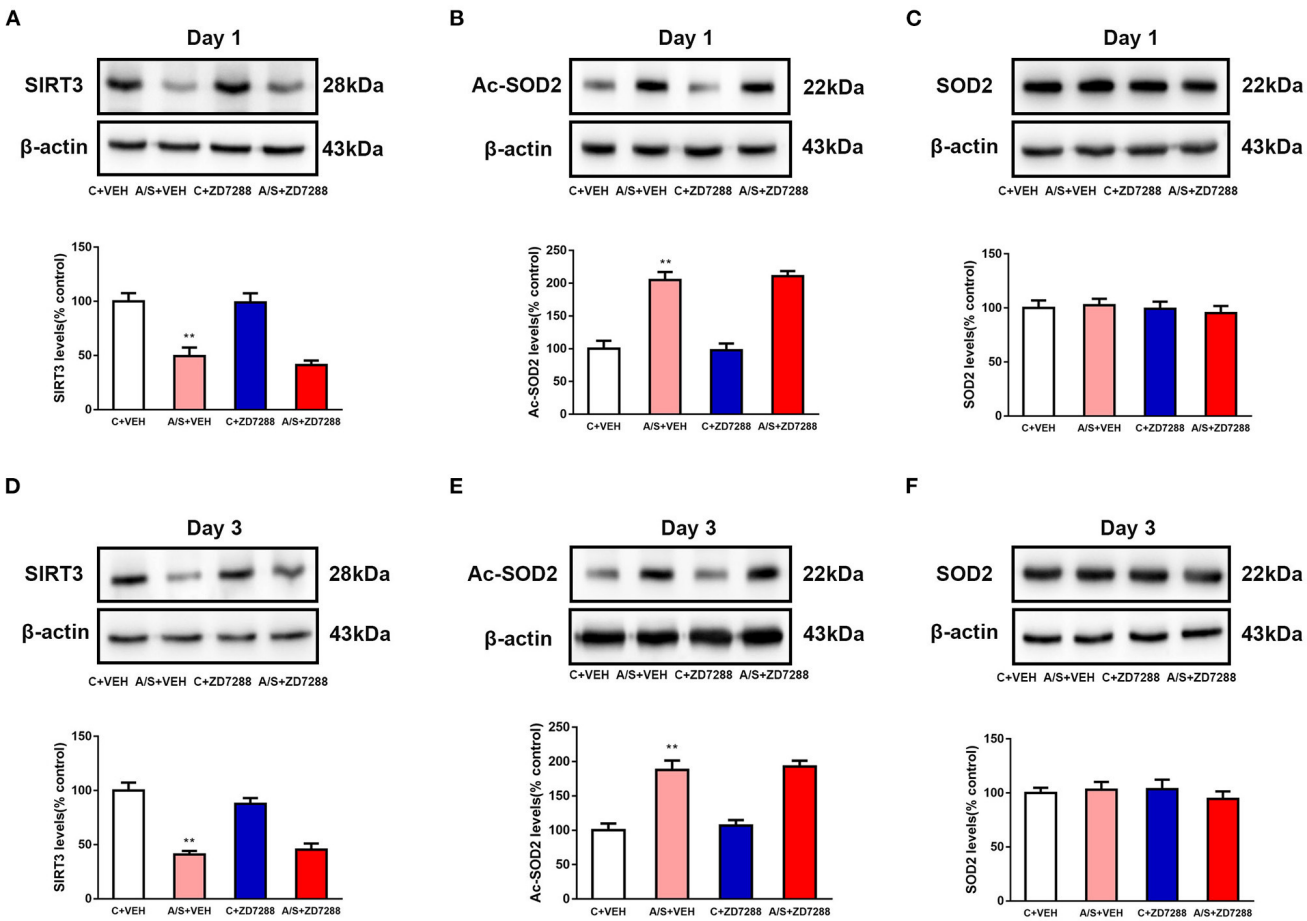
Effect of the HCN1 inhibitor ZD7288 on SIRT3, SOD2, and Ac-SOD2 levels. **(A–F)** SIRT3, Ac-SOD2, and SOD2 expression among the four groups on days 1 and 3 are shown by western blotting and quantification (*n* = 6 per group, **p < 0.01). Data are presented as the means ± SEM.

To clarify the upstream/downstream relationship between HCN1 and SIRT3/SOD2, SIRT3 expression was measured after ZD7288 and anesthesia/surgery interactive treatment. As we can see in Figures 6A,D, the SIRT3 expression was decreased in A/S + VEH than in C + VEH group, but not significantly changed in group A/S +ZD7288 (^**^p < 0.01, Figures 6A,D). Next, the ac-SOD2 level was meaningfully raised in A/S + VEH than in C + VEH group at day 1 and 3 postoperatively (^**^p < 0.01, Figures 6B,E), but not meaningfully changed in A/S +ZD7288 group than in A/S + VEH group. Lastly, the SOD2 level was still not significantly changed among 4 groups day 1 and 3 after anesthesia/surgery (Figures 6C,F). These results suggested that the HCN1 blocker ZD7288 did not prevent anesthesia/surgery-induced SIRT3 decrease and activated SOD2 acetylation in mice mPFC after surgery; hence, HCN1 might be the downstream target in the SIRT3/ac-SOD2 signaling pathway.

### Inhibition of the HCN1 Channel Did Not Affect Mitochondrial Oxidative Stress Reaction Induced by Anesthesia/Surgery in Mice mPFC

**Figure 7:**
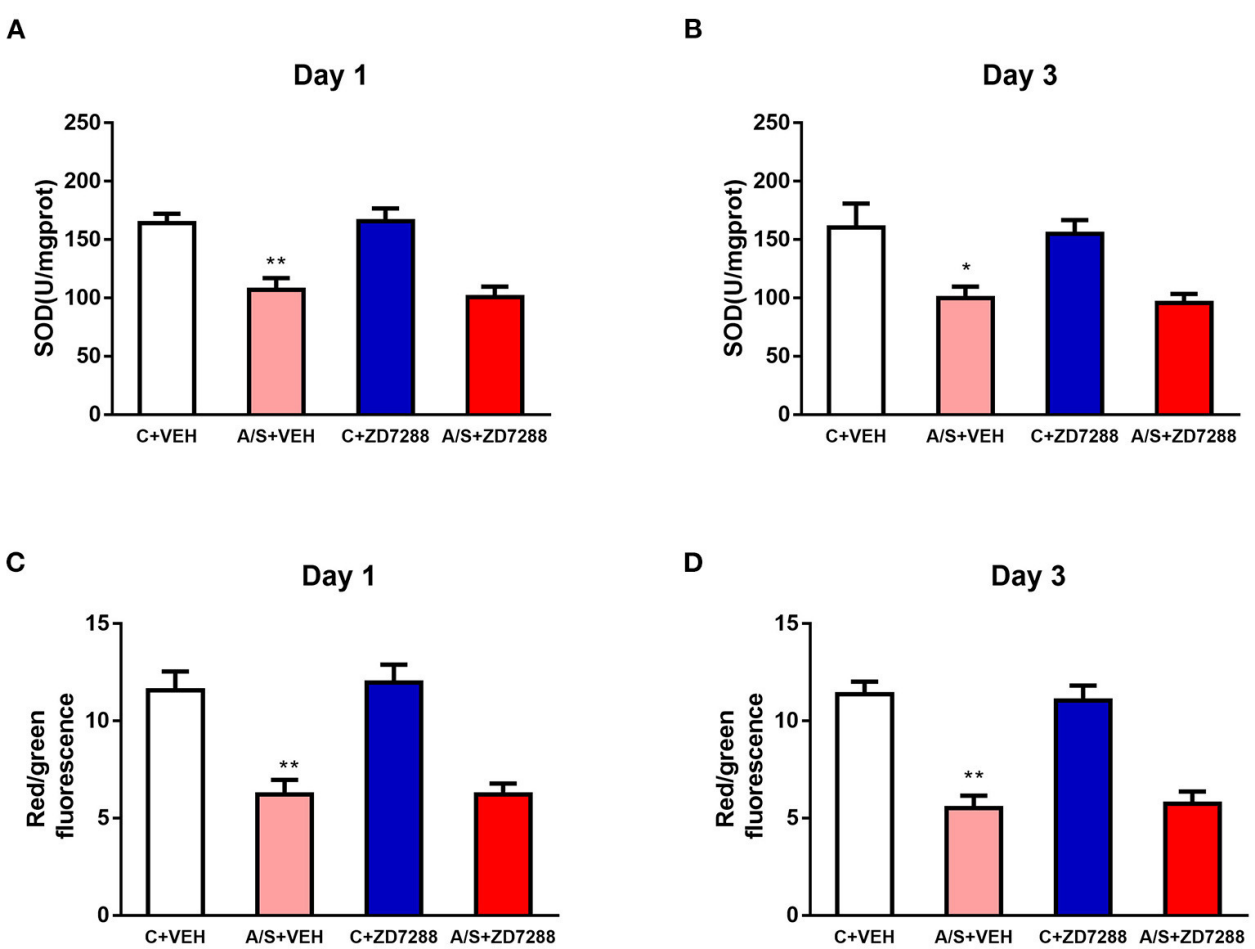
Effect of HCN1 inhibitor on mitochondrial oxidative levels. **(A–D)** Analysis of SOD2 and MMP in the mPFC region among the four groups (*n* = 6 per group, *p < 0.05; **p < 0.01). Data are presented as the means ± SEM.

To observe the interaction between HCN1 and SIRT3-related pathways, the mitochondrial oxidative stress response was measured after ZD7288 treatment. Anesthesia/surgery induced decreased SOD2 and MMP level in A/S + VEH in comparison with C + VEH group in mice mPFC region on day 1 and 3 postoperatively. However, HCN1 blocker ZD7288 did not attenuate the decreased SOD2 and MMP level in A/S + ZD7288 in comparison with A/S + VEH group (^**^p < 0.01, ^*^p < 0.05, Figures 7A–D). These data indicated that inhibition of HCN1 channel activation did not affect SIRT3-related mitochondrial oxidative stress reaction induced by anesthesia/surgery in mice mPFC, also supporting that HCN1 might be the downstream target of the SIRT3/ac-SOD2 signaling pathway.

### Inhibition of the HCN1 Channel Attenuated Anesthesia/Surgery-Induced Anxiety-Like Behavior in Mice

**Figure 8:**
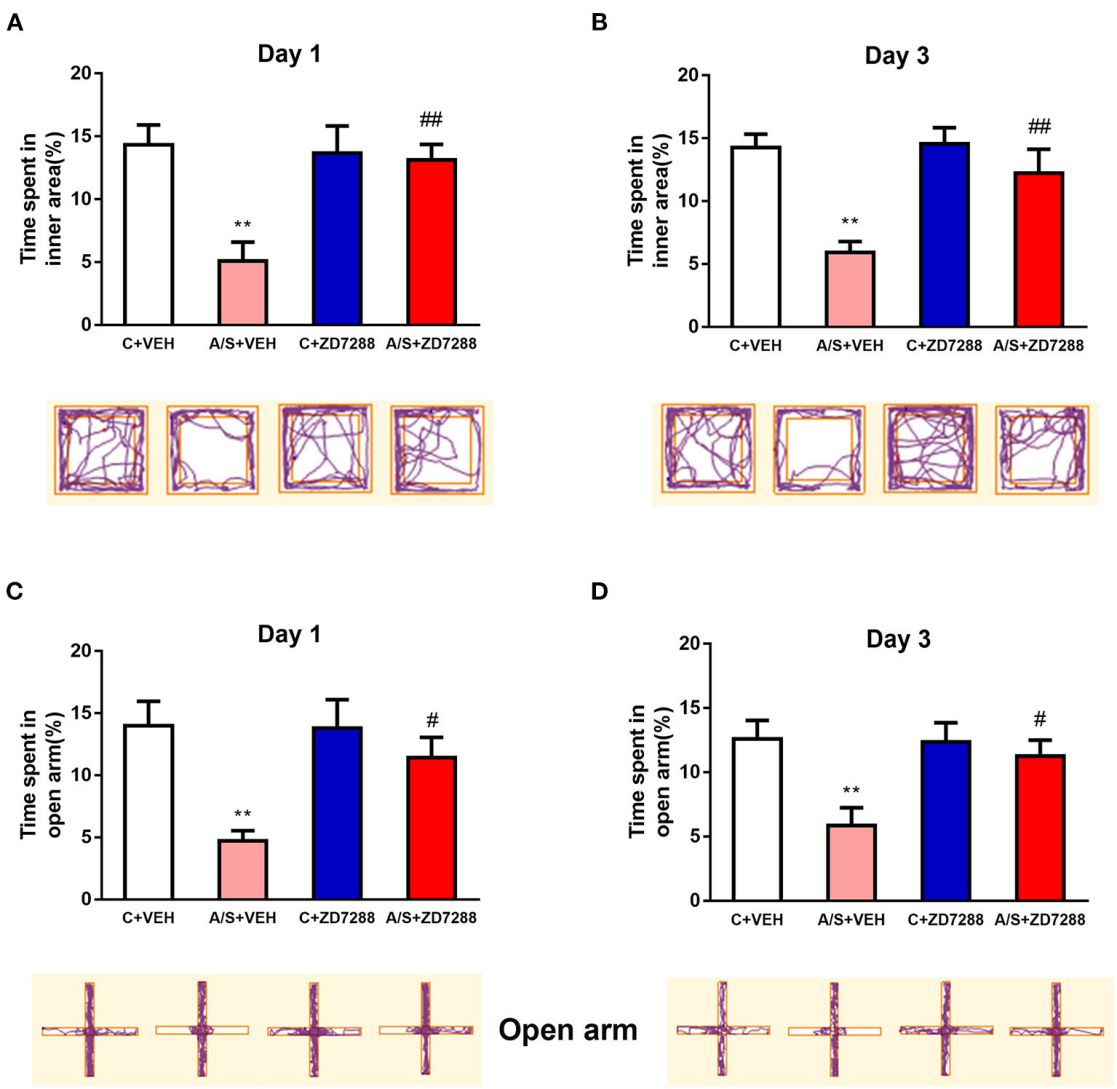
Effect of the HCN1 inhibitor on anxiety-like behavior induced by anesthesia/surgery. **(A,B)** The percent time spent in inner area and movement trace of the OFT among the 4 groups. **(C,D)** The percent time in open arm and movement trace of the EPM among the 4 groups (*n* = 10 per group, *p < 0.05; **p < 0.01; ^#^*p* < 0.05; ^*##*^*p* < 0.01). Data are presented as the means ± SEM.

To demonstrate the role of the HCN1 channel in anxiety emotion after surgery, the OFT and EPM test were used for behavioral testing. In the OFT, the percentage of time in interior region in group A/S + VEH was significantly lower (^**^p < 0.01, Figures 8A,B). Moreover, the HCN1 blocker protected against the decreased percentage of time that was spent in OFT's interior region in group A/S + ZD7288. (## *p* < 0.01, Figures 8A,B). Next, the percentage of time that was spent in EPM's open arm was also significant diminished in A/S + VEH (^**^p < 0.01, Figures 8C,D). In addition, HCN1 blocker protected against the diminished percentage of time that was spent in EPM's open arm in group A/S + ZD7288 (# *p* < 0.05, Figures 8C,D). These consequences showed that HCN1 blocker ZD7288 attenuated anesthesia/surgery induced anxiety emotion in mice.

## Discussion

This study was conducted to examine the effects of SIRT3 on anxiety-like behavior induced by anesthesia/surgery in mice and assess the role of HCN1 channels. We found that SIRT3 attenuated postoperative anxiety by regulating SOD2 acetylation and mitochondria-mediated oxidative stress. The protective effects of SIRT3 on anxiety-like behavior appeared to be related to HCN1 channel function.

Anxiety disorders occur worldwide. In 2019, *Lancet Psychiatry* published an important epidemiological survey by Chinese mental health organizations, which showed that anxiety disorder is currently the most prevalent mental disorder in China (38). The prevalence ranking of mental disorders (including some neurological diseases) from 30 European countries also showed that anxiety disorder was the most common mental disorder, and its prevalence was much higher than that of insomnia, posttraumatic stress disorder (PTSD), Alzheimer's disease, and depression (39). However, patients with anxiety often complain of physical symptoms, such as pain or sleep disturbances, and the rate of missed diagnosis or misdiagnosis is high. Perioperative factors often aggravate anxiety, and most perioperative patients experience anxiety. However, perioperative anxiety, especially postoperative anxiety, has not received enough attention.

Postoperative anxiety, as a perioperative neuropsychiatric symptom, may lead to delayed discharge, chronic postoperative pain, increased delirium, and cognitive impairment and may affect the quality of life and long-term prognosis of patients (40, 41). According to a previous study, the incidence of postoperative anxiety ranges from 4.1% to 79.6%; it seriously affects the postoperative recovery of patients and the time to return to work and is related to postoperative analgesic abuse, chronic pain, and pathological behavior (42). A previous meta-analysis showed that postoperative anxiety might last for >1 year in patients undergoing cardiac surgery and that perioperative anxiety was significantly associated with late postoperative mortality (43). Postoperative anxiety was also associated with the incidence of postoperative atrial fibrillation in patients undergoing coronary artery bypass grafting (44). In a meta-analysis of 27 clinical studies related to postoperative intensive care unit (ICU) anxiety status in 2880 patients, the incidence of anxiety disorders at 2–3 months, 6 months, and 12–14 months after enrolment was 32%, 40%, and 34%, respectively. Anxiety increases the incidence of post-ICU delirium, depression, and PSTD and reduces the quality of life of patients (45). The commonly used acute antianxiety drugs are benzodiazepines, which may aggravate the side effects, such as delirium, and are limited as postoperative antianxiety drugs (9). Venlafaxine, recommended as a first-line anxiolytic, which needs approximately a week to take effect, may not be suitable for the postoperative period (46). Therefore, exploring the mechanism of postoperative anxiety and finding effective and safe agents is of great clinical significance and social value.

The relationship between anxiety and pain has been debated. Preoperative administration of midazolam can reduce postoperative pain scores and the occurrence of postoperative anxiety (47). In a previous study, the dosage of postoperative patient-controlled analgesia pump for patients with anxiety was significantly increased with a decrease in mechanical pain threshold (48). Unmanaged pain can aggravate anxiety, but some studies have found no correlation between anxiety and postoperative pain (49). In the incision pain model, the mechanical pain threshold was reduced 24 h after surgery and gradually returned to normal, but anxiety-like behavior persisted for 5 days after surgery (34), suggesting that the negative emotional anxiety associated with postoperative pain might have a more serious and lasting impact than pain itself. In our study, we also treated the mice with 2% lidocaine cream to relieve postoperative pain, although we could not completely rule out the effect of postoperative pain, we found anxiety symptoms in mice at least 3 days after anesthesia/surgery.

SIRT3, an NAD-dependent deacetylase, is mainly distributed in mitochondria-rich tissues and organs and regulates mitochondrial protein function through acetylation (21). SIRT3 is associated with anxiety in mice with Alzheimer's disease (50). Synaptic electrical activity and neurotransmitter transmission depend on mitochondrial energy supply. Increased SIRT3 expression in hippocampal neurons enhances the release of excitatory neurotransmitters (51). In a spinal cord ischemia model, MMP loss and neuronal apoptosis were protected by ZL006 by stimulating SOD2 deacetylation and mitochondrial enzyme activities in a SIRT3-dependent manner (52). Melatonin relieves contrast-induced acute kidney injury by activating SIRT3 and diminishing ac-SOD2 K68, thus exerting an antioxidative effect (53). Here, we showed that SIRT3 attenuated anesthesia/surgery-induced anxiety-like behavior in mice mPFC via the ac-SOD2 K68-mediated mitochondrial oxidative stress pathway.

Antianxiety behavior associated with the brain-derived neurotrophic factor-mammalian target of rapamycin signaling pathway was revealed by knockdown of HCN1 channels in the hippocampal dorsal CA1 region, suggesting that HCN1 protein might be a target for anxiety disorder treatment (14). In withdrawal anxiety of ethanol dependence disorder, HCN1 expression was increased with changes in the synaptic ultrastructure (11). Ketamine affects HCN1 expression in the prefrontal cortex to alleviate PTSD-associated anxiety symptoms (12). In mice with chronic stress, the HCN1 channel in the basolateral amygdaloid nucleus has been shown to be an important part of the pathophysiology of anxiety and a potential target for new therapies for anxiety (54). In our study, the HCN1 inhibitor ZD7288 could prevent anesthesia/surgery-induced anxiety-like behavior, indicating the key role of the HCN1 channel. However, ZD7288 did not affect SIRT3/ac-SOD2 expression. Additionally, SIRT3 overexpression decreased HCN1 expression, suggesting that the HCN1 channel might be the downstream target of the SIRT3/ac-SOD2 pathway. Our results revealed that SIRT3 might have an important regulatory function in the HCN1 channel through the SOD2 acetylation-mediated mitochondrial pathway in a mouse model of postoperative anxiety.

Our current research has several limitations. First, we only evaluated the mPFC region in our study; thus, we could not rule out the effect of other brain regions in anesthesia/surgery-induced anxiety symptoms. Second, in this study, a separate anesthesia control group was not included because patients undergoing surgery will receive all these steps, and several factors may play a role in the clinical management of such cases. The role of other perioperative factors associated with the SIRT3 and HCN1 channels in the mPFC in postoperative anxiety behavior should be explored in the future studies. Third, the electrophysiological mechanism of the HCN1 ion channel in anesthesia/surgery-induced anxiety behavior was not explored, which should be studied in further studies. Fourth, HCN1 channels have been established to be predominantly localized in neurons in the mouse cortex and rat hippocampus (55, 56). In the transient ischemia model, HCN1 was transiently and markedly increased at 6 h but barely detected at 4 days in the CA1 pyramidal neurons after transient global cerebral ischemia (tgCI), however, HCN1 was expressed in pericytes and astrocytes in the ischemic CA1 subfield 4 days after tgCI. Therefore, HCN1 channel might be a common feature of reactive astrocytes at later stages after ischemic brain injury and these channels might play an important role in the regeneration of ischemic tissue. In hence, we cannot totally rule out the HCN1 channel in glial cell, we may perform the study of HCN1 channel in specific cell type and different time point in the further.

## Conclusions

This study suggest that SIRT3 may ameliorate anesthesia/surgery-induced anxiety-like behavior by preventing SOD2 acetylation-mediated mitochondrial oxidative stress and HCN1 channel dysfunction in the mPFC of mice. SIRT3 may be an expected target for the treatment and diagnosis of postoperative anxiety disorders.

## Data Availability Statement

The raw data supporting the conclusions of this article will be made available by the authors, without undue reservation.

## Ethics Statement

The animal study was reviewed and approved by Xuzhou Medical University.

## Author Contributions

H-HM, QL, C-HZ, and Y-QW: designed the study. QL, NW, Y-PL, CC, H-BW, HH, W-FW, J-TL, Y-KQ, and C-WZ: performed the experiment and collected and analyzed data. H-HM and QL: drafted the manuscript. All authors have read the article and approved the manuscript.

## Funding

This work was supported by Beijing Municipal Administration of Hospitals' Youth Program (QML20200102), National Natural Science Foundation of China (81701040 and 82071180), Natural Science Foundation of Beijing (7212023) awarded to H-HM, and National Natural Science Foundation of China (82171191) awarded to Y-QW.

## Conflict of Interest

The authors declare that the research was conducted in the absence of any commercial or financial relationships that could be construed as a potential conflict of interest.

## Publisher's Note

All claims expressed in this article are solely those of the authors and do not necessarily represent those of their affiliated organizations, or those of the publisher, the editors and the reviewers. Any product that may be evaluated in this article, or claim that may be made by its manufacturer, is not guaranteed or endorsed by the publisher.
